# Editorial: Food tourism: culture, technology, and sustainability

**DOI:** 10.3389/fnut.2024.1390676

**Published:** 2024-05-31

**Authors:** Nassim Naderi, Nona Naderi, Huey Chern Boo, Kuan-Huei Lee, Po-Ju Chen

**Affiliations:** ^1^Independent Researcher, Saint Hyacinthe, QC, Canada; ^2^Université Paris-Saclay, CNRS, Laboratoire Interdisciplinaire des Sciences du Numerique, Orsay, France; ^3^YTB (Singapore) Pte Ltd, Singapore, Singapore; ^4^Singapore Institute of Technology, Singapore, Singapore; ^5^Department of Hospitality, Hotel Management and Tourism, Texas A&M University, College Station, TX, United States

**Keywords:** sustainable tourism, sustainable food and agriculture policy, sustainable diet, innovation foods, sustainable food tourism, environmental sustainability, green packaging, green purchasing behavior

Food tourism (FT) offers an appealing opportunity for promoting sustainable diets, as it attracts visitors to explore local food cultures, products, and activities. It benefits the economy and the environment but faces challenges from new technologies and consumer preferences. One solution is the circular economy which emphasizes waste minimization and resource maximization. The Internet and web application also influence FT through enabling visitors to learn and share food experiences.

Eco-tourism is a form of sustainable tourism that respects and protects nature and culture. It creates jobs and income while conserving resources. The main goal of this Research Topic was to expand and update the current literature on food in eco-tourism, food choice, eating behaviors and sustainability. It explored how food-related decisions and practices affect the environment, society and culture in different contexts and settings. Several researchers have contributed their interesting opinions and findings in the form of reviews and empirical papers to reflect current ethos and concerns in the sphere of food consumption and tourism modernization.

Non wood forest products (NWFP), such as food, medicine, and fibers, are biological goods from forests and trees, valuable for rural and indigenous communities and the environment. For that, Purwestri et al. researched NWFPs preferences in the Czech Republic, aiming to identify opportunities for enhancing their use for livelihoods and environmental conservation. They found two groups of NWFPs based on respondents' choices and suggested policy implications for different forest types, owners, and visitors. They argued that promoting NWFPs can benefit the local bioeconomy and the forest ecosystem while respecting public access rights and national forest policy.

FT is influenced by both the gastronomic identity of a place and the tourists who visit it. Within the scope of our Research Topic, Kalenjuk Pivarski et al. identified the gastronomic factors that shape FT in some of the tourist destinations in Serbia and Bosnia and Herzegovina. The study explores how the local cuisine, food culture, and gastronomic heritage contribute to the sustainable diet, destination attractiveness and competitiveness for food tourists. Local gastronomy and FT are two interrelated concepts that refer to the exploration and enjoyment of the culinary culture and traditions of a destination. Local gastronomy reflects the history, identity, and diversity of a place, while FT allows tourists to enjoy and appreciate it. The behavior and intention of tourists toward consuming local food are influenced by their culture, preferences, health, curiosity, and ethical or environmental values. Local food can be a way of experiencing the destination's culture, identity, and authenticity, or a challenge to overcome one's fears and biases. Mohammadian Pouri et al. suggested travelers' interest in local cuisines can be elevated by reducing perceived risks and improving knowledge, attitudes, and confidence.

Environmental factors can influence eating behavior by affecting the type, amount, and frequency of food intake, as well as the preferences and choices of individuals and groups. The eating behavior of youths is an important factor that influences their weight status and health outcomes. Therefore, understanding the determinants and consequences of eating behavior among youths is essential for developing effective interventions and policies to curb obesity issues. Eating behavior can be influenced by food-related factors (i.e., availability, accessibility, cost, and convenience), marketing and promotion, and cultural and family values. Abang Brian et al. synthesized the approaches, topics, and determinants of dietary patterns among Malaysian this population. Focusing on the Malaysian youths from different ethnic backgrounds and regions, the researchers underscored a literature gap in the specific food choices, eating patterns and weight management issues. Complementing most of the quantitative past research, qualitative studies are necessary to explore the complex interactions among personal, social, and environmental factors.

The ongoing shifts in food consumption patterns due to various influences such as culture, economy and ecology require regular research updates. It is vital to communicate effectively with policymakers and other stakeholders about the best ways to improve eating behavior. Legislation can also play an important role in influencing consumers' food choices and promoting healthier diets. Alangari discussed the Saudi Arabia Nutrition Labeling Policy, a new initiative by the SFDA to promote healthy eating habits among consumers. The policy requires restaurants to display the calorie content on their menus and caloric information on their food delivery applications, so that customers can make informed decisions about their choice. Researcher investigated how restaurants on these applications adhere to this policy, offering valuable insights for decision-makers to strengthen the enforcement of calorie information policies on online food ordering platforms, restaurant webpages, social media, and advertising campaigns.

Labeling and transparent packaging (TP) are important in FT to influence consumers' perception, trust, and satisfaction with the products purchased. Labeling and TP can also enhance positive image and reputation, allowing food producers and destinations to increase their competitiveness and profitability in the global market. Kuang et al. examined how TP for organic foods influences tourists' green purchasing behavior. Surveying individual tourists in a popular eco-tourism destination, the researchers found that TP increased the perceived quality, freshness, and environmental friendliness of organic foods, which positively affected tourists' willingness to pay more and intention to repurchase subsequently. Hence, TP can be an effective marketing strategy for organic food producers and retailers to attract eco-conscious tourists (Kuang et al.). Green packaging (e.g., biodegradable, recyclable, reusable, smart packaging) is a way of reducing the environmental and social costs of packaging food by using materials and methods that save resources, prevent pollution, protect food quality and safety. It does not only enhance eco-tourism experience but reflects the environmental and social values of the tourism sector and the consumers. FT can positively impact the economy, society and environment aspects if executed in a responsible and sustainable manner. Technology can play a key role in FT by providing information, communication, and innovation tools that enhance the quality and accessibility of food experience. Overall, the present Research Topic collectively covers a range of perspectives, methods, and insights from experts in the field. Readers can explore some examples of how FT and culture, technology, and sustainability interact in different contexts. They can also identify the challenges and opportunities for the future of FT and culture.

## Author contributions

NaN: Writing – original draft, Writing – review & editing. NoN: Writing – review & editing. HB: Writing – review & editing. P-JC: Writing – review & editing. K-HL: Writing – review & editing.

